# Association between SMOFlipid and impaired brain development on term-equivalent age brain magnetic resonance imaging in very preterm infants

**DOI:** 10.1186/s12887-024-05153-8

**Published:** 2024-10-29

**Authors:** Mountasser M. Al-Mouqdad, Belal Alshaikh, Haider H. Sumaily, Nabeel A. Alodhaidan, Latifah AlMahmoud, Ameen A. Almotiri, Mousa A. Alkhourmi, Mazen M. Abounassif, Ahmed F. Beh, Mashael A. Alawad, Amani A. Albraiki, Aziza A. Alqarni, Maha R. Al-Anazi, Nadia A. Basodan, Fuddah M. Assiri, Suzan S. Asfour

**Affiliations:** 1 Neonatal Intensive Care, Hospital of Pediatrics, King Saud Medical City, Al Imam Abdul Aziz Ibn Muhammad Ibn Saud, Riyadh, 12746 Saudi Arabia; 2 Department of Pediatrics, Cumming School of Medicine, University of Calgary, Calgary, AB Canada; 3 Pediatric Gastroenterology Department, Hospital of Pediatrics, King Saud Medical City, Riyadh, Saudi Arabia; 4 Radiology Department, Hospital of Pediatrics, King Saud Medical City, Riyadh, Saudi Arabia; 5 General Pediatrics Department, Hospital of Pediatrics, King Saud Medical City, Riyadh, Saudi Arabia; 6 Pharmacy Department, Pharmaceutical Care Services, King Saud Medical City, Riyadh, Saudi Arabia; 7 Clinical Pharmacy Department, Pharmaceutical Care Services, King Saud Medical City, Riyadh, Saudi Arabia

**Keywords:** Brain morphology, Growth, Lipid emulsion, Magnetic resonance imaging, Parenteral nutrition, Premature infant

## Abstract

Soybean oil, medium-chain triglycerides, olive oil, and fish oil (SMOFlipid) is used without evidence of benefits. We investigated the relationship between lipid emulsions and brain injury in term-equivalent age magnetic resonance imaging (MRI) in 148 very preterm infants with a birth weight of < 1500 g at ≤ 32 gestational weeks in a neonatal intensive care unit. Infants who received soybean-based lipid emulsions between January 2015 and December 2018 were compared with those who received SMOFlipids between January 2019 and December 2022. A negative binomial generalized linear model was applied for bivariate analysis. Modified log-Poisson regression with generalized linear models and a robust variance estimator (Huber–White) were applied to adjust for potential confounders. The Kidokoro score was used to determine if lipid emulsion type would affect brain morphology and growth at term-equivalent age. Eighty-six (58.9%) received SMOFlipid. SMOFlipid was associated with lower focal signal abnormality, myelination delay, increased extracerebral space, and cerebellar volume reduction (*P* = 0.02, *P* = 0.007, *P* = 0.01, *P* = 0.02, respectively). SMOFlipidis are associated with brain insult, especially in white matter, cortical gray matter, and the cerebellum. Well-designed studies are needed to investigate the effect of lipid emulsions on the central nervous system.

## Introduction

Advanced neonatal-perinatal medicine techniques have improved the survival of premature infants worldwide. However, this improvement has been accompanied by an increase in major neonatal morbidities [1]. Among the strategies available for reducing neonatal morbidity, improving early nutrition is among the most promising [2]. Several recent studies have concluded that optimizing nutrition in the first few days following birth is important and safe. However, many babies continue to have less than optimal postnatal growth.

It is the standard of care in the first days of life for preterm infants to receive parenteral nutrition (PN) [3]. Despite uncertain relative impacts, energy, protein, and lipid intake positively correlate with cognitive ability [4]. Up to 50% of energy comes from intravenous lipids, essential fatty acids, and long-chain polyunsaturated fatty acids [5]. Recent studies have found that sufficient concentration of DHA is associated with better maturation of gray and white matter and improved cerebral and cerebellar microstructure development [6]. Further studies showed that higher lipid intake in the first two weeks in preterm babies decreased brain MRI abnormalities at a term-equivalent age [7].

Intralipid^®^ and SMOFlipid^®^ [**S**oybean oil 30%, **M**edium-chain triglycerides 30%, **O**live oil 25%, and **F**ish oil (omega-3) 15%] are the most used intravenous lipid emulsions worldwide. Intralipid contains mainly soybean oil 100%, which is crucial for tissue, retinal, and brain development, as it is a rich source of omega-6 polyunsaturated fatty acids (ῳ-6PUFA). However, it leads to intrahepatic cholestasis, promotes inflammation, and causes immune system suppression [8–10].

SMOFlipid is used despite limited evidence. Our recent study found SMOFlipid was associated with lower bronchopulmonary dysplasia (BPD). However, it was associated with a higher frequency of late-onset sepsis (LOS) and delayed growth velocity [11]. A new study reported no alteration in BPD and mortality in premature infants receiving SMOFlipid during their hospital stay [12]. Another report found no difference between Intralipid and SMOFlipid in terms of neurodevelopmental outcome [13].

The greatest increase in volume and maturation of the cerebrum, cerebellum, and brain stem occurs in the third trimester of pregnancy [14]. PN plays a crucial role in this stage. Despite increasing use of SMOFlipid and emerging evidence, there remains a lack of consensus regarding its efficacy and safety compared to intralipid. Without knowing which type of intravenous lipid emulsion is best and most appropriate for this growth phase, it is necessary to investigate the influence of both types on brain volume and maturation.

We evaluated the impact of Intralipid and SMOFlipid on white matter, cortical and deep gray matter, and the cerebellum. Brain growth was scored using the Kidokoro scoring system in babies born at less than 32 weeks gestation who underwent MRI at term-equivalent age [15]. We hypothesized that both lipid emulsions positively and negatively affect brain tissue.

## Materials and methods

### Study design

This retrospective chart review included a cohort of preterm infants admitted to the NICU at King Saud Medical City (KSMC) tertiary-care referral center between July 2014 and December 2022. Including level 3, the NICU at KSMC has an average annual admission of 1,100 patients. This study complied with the Declaration of Helsinki and Good Pharmacoepidemiology Practice guidelines and was approved by the medical ethical review committee of King Saud Medical City (KSMC-reference number H1RI-25-Feb19-01), which also approved waived informed consent.

### Inclusion and exclusion criteria

We included infants at KSMC at ≤ 32 weeks of gestation with a birth weight of < 1,500 g, admitted to the NICU, and who underwent brain MRI at term-equivalent age. All infants who received total PN plus lipid emulsion (LE) within the first 24 h of birth were included. Infants with major congenital anomalies, those who were not born at KSMC or transferred out, with inborn errors of metabolism, who did not receive PN, and those whose data could not be retrieved were excluded.

### Data collection and follow-up

The infants’ charts from the NICU admission until discharge or death were retrieved. Participants’ demographic, clinical, and outcome data were reviewed. Maternal data, including antenatal steroid treatment, mode of delivery, gestational diabetes mellitus, and maternal hypertension were also collected.

### Study outcome

The primary outcome was the effect of SMOFlipid on brain morphology and growth in brain MRI at term-equivalent age in preterm infants who were ≤ 32 weeks gestational age. Brain growth was scored using the Kidokoro scoring system [15].

### Definitions

#### Nutrition protocol

Total parenteral nutrition (TPN) Protocol: TPN was started after birth using starter TPN. Individualized TPN was written on daily basis. Starter PN includes dextrose 10%, amino acids 4%, and calcium gluconate 0.01 mmol/mL. Individualized TPN bags containing amino acids, dextrose, minerals, trace elements, water-soluble vitamins, and fat-soluble vitamins were initiated within the first 24 h of life and infused for 24 h. The infants were categorized into two LE groups: (1) SMOFlipid (Fresenius Kabi, Melrose Park, IL, USA). Infants were given parenteral multi-oil emulsions containing soybean oil, MCT, olive oil, and fish oil, and (2) Intralipid 20% medium-chain and long-chain fat injection containing soybean oil and MCT. The initial LE dose was 0.5 to 1.0 g per kg per day soon after birth. It was increased by 0.5 to 1.0 g per kg per day every 24 h with a maximum of 3.0 g to 3.5 g per kg per day. The level of triglyceride was not measured in the routine investigation. Enteral nutrition was started as soon as possible after birth. Expressed breast milk or preterm formula was administered through an orogastric tube based on the feeding protocol, which is dependent on birth weight.

A preterm formula was administered when human milk was not available or sufficient. TPN was stopped when enteral feeding reached 100 ml per kg per day.

#### Growth anthropometry

Body weight and head circumference (HC) were recorded on admission to the neonatal unit. NICU nurses measured body weight and HC. Body weight was measured with an electronic scale calibrated to 0.05 kg. Head circumference was measured weekly with a measuring tape, which is precise to the nearest millimeter. The change in body weight was recorded daily, and HC weekly. The 2013 Fenton growth chart was used for the z-score calculation [16].

#### Term-equivalent MRI

All participants underwent MRI without sedation. MRI was performed using a GE Optima MR450w 1.5-T, 70-cm scanner (General Electric Co., Windsor, CT, USA). Three-dimensional spin-echo T1-weighted images, axial and coronal T2-weighted images, axial fluid-attenuated inversion recovery images, and diffusion- and susceptibility-weighted images were obtained. The standardized scoring system developed by Kidokoro et al. were used to evaluate the images [17]. It identifies the global and regional alterations in brain structure by measuring precise quantitative biometrics. Recent studies have reported that the Kidokoro score is reliable for detecting short- and long-term neurodevelopmental outcomes in premature infants [17]. Additionally, another study examined the predicative validity, inter- and intrarater reproducibility for the neurodevelopmental outcomes. They found the score is valid in predicating the Neuro-Sensory Motor Developmental Assessment [18–20]. This scoring system assesses abnormalities in the cerebral white matter (WM), cortical gray matter (GM), deep GM, and cerebellum [17]. Cerebral WM abnormality was assessed using six items graded between 0 and 4: (1) cystic degeneration, (2) focal signal abnormalities, (3) delayed myelination, (4) corpus callosum thinning, (5) lateral ventricular dilatation, and (6) WM-volume reduction. Cortical GM abnormality was assessed using three items graded between 0 and 4: (1) signal abnormality, (2) delayed gyration, and (3) extracerebral cerebrospinal fluid space dilatation. Deep GM and cerebellar abnormalities were assessed using two items graded between 0 and 4: (1) signal abnormality and (2) volume reduction. The total scores for each area were calculated separately [17]. Each region was categorized as having no abnormality and mild, moderate, or severe abnormalities [17]. A global brain abnormality score was calculated by summing the four regional total scores. Infants were then classified as normal or having mild, moderate, or severe brain injury based on Kidokoro score.

The images were interpreted by a neuroradiologist blinded to all clinical data except birth weight and gestational age.

### Statistical analysis

Before performing the analysis, we checked for missing data. Data were analyzed using SPSS version 26 (IBM Corp., Armonk, NY).

Data regarding infants and maternal variables are presented using descriptive statistics, including frequency, percentage, median, and interquartile range. The Kolmogorov–Smirnov test and a visual inspection of histograms were performed to determine the distribution of quantitative variables. The Mann–Whitney U test was used for between-group comparisons of ordinal variables and continuous variables. Fisher’s exact test was used to determine an association between categorical variables.

To analyze the association between type of lipid emulsion and outcomes, we first conducted a univariate relative risk analysis on the recorded variables (gestational age; birth weight; gender; small for gestational age; 1 and 5 min Apgar score; necrotizing enterocolitis; surfactant use; total invasive ventilator days; severe intraventricular hemorrhage; bronchopulmonary dysplasia; patent ductus arteriosus required treatment; maternal hypertension; antenatal and postnatal steroid treatment; premature rupture of membrane; sepsis; delivery mode; inotropes, dextrose intake; amino acids; LE and days of TPN) because we considered them to be potential confounders. All variables that were significant in the univariate analysis were included in the final multivariable regression model. A negative binomial generalized linear model was applied for bivariate analysis. Modified log-Poisson regression with generalized linear models and a robust variance estimator (Huber–White) were applied to adjust for potential confounders. All statistical tests were two-tailed. P-values of < 0.05 were considered statistically significant.

## Results

**Fig. 1:**
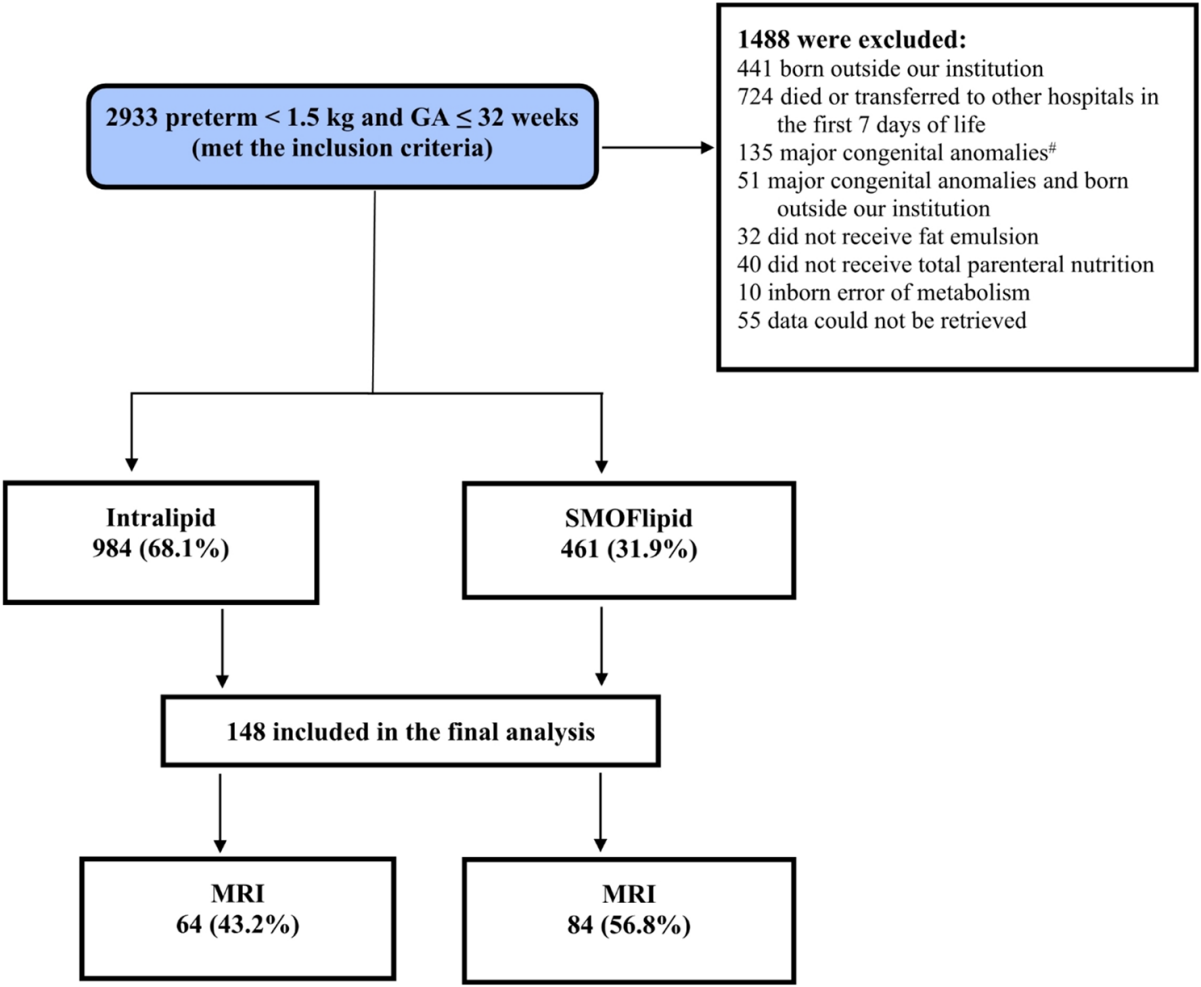
Flow chart of patient selection. *GA* gestational age, *MRI* magnetic resonance imaging

During the study period, 2364 preterm infants with ≤ 32 gestational weeks and a birth.

weight of < 1500 g were admitted to the NICU (level 3). One thousand and two hundred ninety-seven patients did not have brain MRI. One hundred and forty-eight met the inclusion criteria and were included in the final analysis (Fig. 1).

Among 148 infants, 84 received SMOFlipid, and 64 were in the Intralipid group. Maternal and neonatal characteristics are summarized in Table 1. Apgar scores at 5 min were significantly higher in infants receiving SMOFlipid than in the Intralipid group (*P* = 0.006, Table 1).

**Table 1:** Maternal and neonatal characteristics of the study participants

Variables	Type of lipid emulsion		*P*-value
	Intralipid (*n* = 64)	SMOFlipid (*n* = 84)	
Gestational age, weeks, median (IQR)	28 (26, 31)	29 (26, 31)	0.67
Birth weight, grams, median (IQR)	1015 (875, 1243)	1073 (855, 1303)	0.73
Antenatal steroid, n (%)	29 (45.3)	29 (34.5)	0.23
Male sex, n (%)	42 (65.6)	48 (59.3)	0.49
Chorioamnionitis, n (%)	0 (0)	1 (1.2)	1
Gestational hypertension, n (%)	16 (25)	11 (13.1)	0.09
Small for gestational age, n (%)	10 (15.6)	6 (7.1)	0.12
Premature rupture of the membrane	4 (6.3)	6 (7.1)	1
Gestational diabetes mellitus	3 (4.7)	3 (3.7)	1
Cesarean section, n (%)	42 (66.7)	65 (80.2)	0.08
1 min Apgar score, median (IQR)	5 (3, 6)	5 (3,6)	0.76
5 min Apgar score, median (IQR)	6.5 (5, 7)	7 (6, 8)	0.005*
Use of surfactant, n (%)	46 (71.9)	65 (77.4)	0.45
Patent ductus arteriosus, n (%)	43 (67.2)	52 (61.9)	0.60
Patent ductus arteriosus required treatment, n (%)	9 (14.1)	11 (13.1)	1
Necrotizing enterocolitis, n (%)	31 (48.4)	32 (38.1)	0.24
Intraventricular hemorrhage, n (%)	36 (56.3)	53 (63.1)	0.50
Severe intraventricular hemorrhage, n (%)	26 (40.6)	43 (51.2)	0.24
Pulmonary hemorrhage, n (%)	8 (12.9)	6 (7.1)	0.27
Sepsis, n (%)	6 (9.4)	17 (20.2)	0.11
Inotropes, n (%)	27 (42.2)	34 (40.5)	0.87
Postnatal steroids, n (%)	8 (5.4)	14 (9.5)	0.64
Non-invasive ventilation, n (%)	63 (98.4)	79 (95.2)	0.38
Length of mechanical ventilation (days), median (IQR)	9 (2, 20.5)	8.5 (1, 32.25)	0.86
Bronchopulmonary dysplasia, n (%)	41 (64.1)	56 (71.8)	0.37
Age at magnetic resonance imaging (weeks), median (IQR)	37 (35, 40)	38 (35, 41)	0.34

*Statistically significant at 5% level

Univariate analysis showed a significant difference in the median intake of macronutrients. The average daily lipid and amino acid intake in the first week (g/kg/day) were higher in infants who received SMOFlipid (*P* = 0.007, *P* = 0.005, respectively). In addition, the average daily intake of dextrose in the first week (mg/kg/min) was higher in infants who received Intralipid (*P* < 0.001). However, there is no significant difference in the energy intake (kcal/kg/day) between both groups. In addition, both groups had no significant differences in weight, HC and length measures at the time of discharge and no difference in the z-score for weight, HC, length at the time of discharge (Table 2).

**Table 2:** Nutritional intake and growth anthropometrics of the study participants

Variables	Type of lipid emulsion		*P*-value
	Intralipid (*n* = 64)	SMOFlipid (*n* = 86)	
Average daily dextrose intake in the first week (g/kg/day), median (IQR)	8.38 (7.48–9.45)	7.90 (6.88–8.54)	0.009*
Average daily lipid intake in the first week (g/kg/day), median (IQR)	1.86 (1.4–2.14)	2.14 (1.83, 2.57)	0.001*
Average protein intake in the first week (Kcal/kg/day), median (IQR)	3.83 (3.43–4)	4 (4–4)	< 0.001*
Average energy intake in the first week (Kcal/kg/day), median (IQR)	75.4 (66.56–80.33)	74.9 (70.72–82.43)	0.34
Duration of parenteral nutrition (days), median (IQR)	30 (13–54)	36 (19–60)	0.32
Weight at hospital discharge (gram), median (IQR)	2022 (1847–2250)	2305 (1925–2880)	0.12
Weight Z-score at hospital discharge, median (IQR)	−2.61 (− 5.01–−1.52)	−2.15 (− 3.03–−1.43)	0.33
∆ Weight z-score, median (IQR)	−1.20 (− 2.54–−0.76)	−2.19 (− 3.10–−1.50)	0.03*
Length at hospital discharge (cm), median (IQR)	41.5 (39–45)	45 (41–48)	0.09
Length Z-score at hospital discharge, median (IQR)	−4.49 (− 5.96, − 1.49)	−2.93 (− 4.15, − 0.66)	0.20
Head circumference at hospital discharge (cm), median (IQR)	30.5 (27.25–32.5)	32 (30.5–34.5)	0.05
Head circumference Z-score at hospital discharge, median (IQR)	−2.59 (− 6.78, − 1.16)	−1.08 (− 2.97, − 0.39)	0.08

*Statistically significant at 5% level

Univariate analysis revealed that neonates who received SMOFlipid had abnormal brain MRI maturation and morphology. SMOFlipid administration had poor focal signal abnormality, myelination delay, increased extracerebral space, cerebellar volume reduction, and an increased total cerebellar score (*P* = 0.007, *P* = 0.005, *P* = 0.001, *P* = 0.01, *P* = 0.04, respectively, Table 3).

**Table 3:** Univariate analysis of global brain abnormalities scores on TEA-MRI stratified by the type of parenteral lipid emulsions

Variable	Type of lipid emulsion		*P*-value
	Intralipid (*n* = 64)	SMOFlipid (*n* = 86)	
**White matter score**			
Cystic lesions, median (minimum, maximum)	0 (0–4)	0.5 (0–4)	0.14
Focal signal abnormality, median ( minimum, maximum )	0 (0–3)	1 (0–4)	0.007*
Myelination delay, median ( minimum, maximum )	1 (0–2)	1 (0–2)	0.005*
Thinning of the corpus callosum, median ( minimum, maximum )	0 (0–2)	0 (0–2)	0.88
Dilated lateral ventricles, median ( minimum, maximum )	0 (0–3)	1 (0–3)	0.10
Volume reduction, median ( minimum, maximum )	2 (0–3)	2 (0–3)	0.75
Total white matter score, median ( minimum, maximum )	6 (1–16)	7 (0–15)	0.11
**Cortical gray matter score**			
Signal abnormality, median ( minimum, maximum )	0 (0–4)	0 (0–4)	0.78
Gyral maturation, median ( minimum, maximum )	0 (0–2)	0 (0–3)	0.81
Increased extracerebral space, median ( minimum, maximum )	0 (0–3)	0 (0–3)	0.001*
Total cortical gray matter score, median ( minimum, maximum )	0 (0–8)	0 (0–9)	0.19
**Deep gray matter score**			
Signal abnormality, median ( minimum, maximum )	0 (0–4)	0 (0–4)	0.68
Volume reduction, median ( minimum, maximum )	0 (0–3)	0 (0–3)	0.25
Total deep gray matter score, median ( minimum, maximum )	0 (0–7)	0 (0–7)	0.98
**Cerebellum score**			
Signal abnormality, median ( minimum, maximum )	0 (0, 4)	0 (0, 4)	0.40
Volume reduction, median ( minimum, maximum )	2 (0, 3)	3 (0, 3)	0.01*
Total cerebellar score, median ( minimum, maximum )	2 (0–7)	3 (0–7)	0.04*
**Global brain abnormality score, median (minimum, maximum )**	8 (2–30)	11 (0–38)	0.12

*Statistically significant at 5% level

After adjusting the significant variables in the univariate analysis, the multivariable regression analysis using Modified log-Poisson regression revealed that SMOFlipid administration negatively impacted neurological outcomes compared with Intralipid. Infants who received SMOFlipid had significantly poor focal signal abnormality, myelination delay, increased extracerebral space, and cerebellar volume reduction (*P* = 0.02, *P* = 0.007, *P* = 0.01, *P* = 0.02, respectively, Table 4).

**Table 4:** Multivariable regression of global brain abnormalities scores on TEA-MRI stratified by the type of parenteral lipid emulsions

Variables	Unadjusted coefficient 95% CI	*P*-value	Adjusted coefficient 95% CI	*P*-value
**White matter score**				
Cystic lesions^#^	1.38 (0.92, 2.07)	0.13	1.44 (0.94, 2.22)	0.09
Focal signal abnormality^$^	1.92 (1.22, 3.01)	0.005*	1.73 (1.10, 2.72)	0.02*
Myelination delay^@^	0.74 (0.61, 0.91)	0.004*	0.76 (0.63, 0.93)	0.007*
Thinning of the corpus callosum^^^	1.01 (0.67, 1.51)	0.98	0.90 (0.60, 1.36)	0.62
Dilated lateral ventricles^&^	1.30 (0.92, 1.84)	0.14	1.27 (0.91, 1.77)	0.17
Volume reduction^¥^	1.04 (0.83, 1.29)	0.76	1.05 (0.84, 1.32)	0.66
Total white matter score^€^	1.16 (0.95, 1.41)	0.14	1.12 (0.93, 1.35)	0.23
**Cortical gray matter score**				
Signal abnormality^£^	0.78 (0.35, 1.77)	0.56	0.89 (0.38, 2.09)	0.79
Gyral maturation^µ^	1.10 (0.47, 2.59)	0.82	1.09 (0.47, 2.49)	0.85
Increased extracerebral space^α^	2.97 (1.26, 7.02)	0.01*	2.97 (1.26, 7.02)	0.01*
Total cortical gray matter score^β^	1.22 (0.69, 2.16)	0.49	1.25 (0.69, 2.78)	0.46
**Deep gray matter score**				
Signal abnormality©	1.08 (0.50, 2.35)	0.84	1.08 (0.50, 2.34)	0.84
Volume reduction^∞^	1.40 (0.86, 2.28)	0.17	1.42 (0.86, 2.24)	0.18
Total deep gray matter score°	1.04 (0.61, 1.79)	0.88	0.97 (0.57, 1.65)	0.90
**Cerebellum score**				
Signal abnormality^¢^	1.33 (0.82, 2.17)	0.25	1.30 (0.89, 2.14)	0.31
Volume reduction^¡^	1.22 (1.03, 1.44)	**0.02**	1.22 (1.03, 1.44)	0.02*
Total cerebellar score^§^	1.24(0.99, 1.55)	0.05	1.21 (0.98, 1.50)	0.08
**Global brain abnormality score** ^**¶**^	1.18(0.94, 1.49)	0.16	1.21 (0.98, 1.50)	0.09

*Statistically significant at 5% level

^#^Adjusted coefficient for severe intraventricular hemorrhage and necrotizing enterocolitis

^$^Adjusted coefficient for severe intraventricular hemorrhage

^@^Adjusted coefficient for total invasive ventilator days

^^^Adjusted coefficient for total invasive ventilator days and severe intraventricular hemorrhage

^&^Adjusted coefficient for birth weight, total invasive days, surfactant, bronchopulmonary dysplasia and severe intraventricular hemorrhage

^¥^Adjusted coefficient for bronchopulmonary dysplasia

^€^Adjusted coefficient for total invasive days, surfactant and severe intraventricular hemorrhage

^£^Adjusted coefficient for signal abnormality and apgar one

^µ^Adjusted coefficient for bronchopulmonary dysplasia

^α^Adjusted coefficient for nothing

^β^Adjusted coefficient for Apgar one

©Adjusted coefficient for necrotizing enterocolitis and total invasive ventilator

^∞^Adjusted coefficient for invasive ventilator (days), severe intraventricular hemorrhage and necrotizing enterocolitis

°Adjusted coefficient for invasive ventilator (days), severe intraventricular hemorrhage and necrotizing enterocolitis

^¢^Adjusted coefficient for gestational age, birth weight, surfactant, invasive ventilator (days), bronchopulmonary dysplasia, pulmonary hemorrhage, severe intraventricular hemorrhage and necrotizing enterocolitis

^¡^Adjusted coefficient for nothing

^§^Adjusted coefficient for Birth weight, surfactant, invasive ventilator (days), bronchopulmonary dysplasia, pulmonary hemorrhage, severe intraventricular hemorrhage and necrotizing enterocolitis

^**¶**^Adjusted coefficient for surfactant, invasive ventilator (days), bronchopulmonary dysplasia, severe intraventricular hemorrhage and necrotizing enterocolitis

## Discussion

We found that preterm infants of ≤ 32 weeks gestational age and receiving SMOFlipid as part of total PN during their hospital stay had more significant detrimental effects than Intralipid on brain MRI maturation and morphology at term-equivalent age. Receiving SMOFlipid was associated with more focal signal abnormality in WM, increased extracerebral space in cortical GM, and more volume reduction in the cerebellum. In contrast, Intralipid administration as a LE was associated with more myelination delay in WM. These results were independent of gestational age, birth weight, sepsis, bronchopulmonary dysplasia, and postnatal steroids.

Lipids are highly enriched in the central and peripheral nervous systems and are critical for many essential functions. Prematurity is said to cause a high rate of gray and WM and cortical abnormalities [21, 22]. Therefore, theoretically, the purpose of initiating lipids as an intravenous emulsion in the early days of premature babies’ life is to minimize brain injury and improve neurodevelopment. Intralipid or SMOFlipid improved the short developmental outcome of small pigs [23]. Although SMOFlipid increased the quantity of DHA in red blood cells, the brain structures were unaffected, and there were no improvements in all short-term measurements and physical activity in all groups.

Beauport et al. investigated the impact of early nutritional intake, in general, on brain maturation assessed by MRI scans in a small number of premature infants who were less than or equal to 30 weeks gestational age [7]. They used soybean, and a mixture of soybean and olive oil, introduced at two different times. High energy and lipid intake was associated with improved maturation scores in brain MRI. They did not recognize the relationship between the type of LE and the incidence of brain lesions and dysmaturity. Another study by Caterina Coviello et al. evaluated the effect of early nutritional intake on brain development in premature infants under 31 weeks of gestational age. They found a positive link between increased cumulative fat, enteral protein, fat, caloric intake, and brain component volumes [24]. The type of lipid solution here was only Intralipid 20%.

Regarding the impact of the type of LE on HC, we observed conflicting results in published reports. Costa et al. found in their randomized controlled trial that SMOFlipid mitigates the reduction in HC from birth to 36 weeks postmenstrual age or at discharge [25]. However, Gallini et al. displayed in their randomized controlled trial no difference in HC during the hospital stay and at 12 and 24 months of age between SMOFlipid and Intralipid [13]. We did not find the effect of lipid type on the HC after we adjusted several variables.

Different studies have investigated the relationship between the type of intravenous LE and the neurodevelopmental outcome [13, 26–28]. Thanhaeuser et al. and Gallini et al. found no superiority of Intralipid or SMOFlipid in improving the neurodevelopmental outcome of extremely premature babies at 12 and 24 months of corrected age. However, Torgalkar et al. found a lower probability of neurodevelopmental delay in premature babies who received SMOFlipid. Chen et al. found that children who received SMOFlipid rather than soybean oil-based emulsions at 2 years had lower incidences of epilepsy, cerebral palsy, developmental disorders, and attention-deficit hyperactivity disorder.

According to the above, first, no study has assessed brain maturation and morphology alteration in brain MRI at term-equivalent age as an outcome of initiation of different lipid emulsions in premature babies. Second, all the studies have concluded that there is uncertainty about the priority, efficacy, and safety of specific lipids in premature infants needing PN. Different populations, supplements, and preparation methods may play a role in this undefined status.

Some limitations to this study should be considered. The retrospective design and observational nature of the study may lead to confounding bias. Additionally, brain MRI is not routinely performed for all premature infants receiving parenteral nutrition (PN) at term-equivalent age, and the decision to perform MRI was left to the discretion of the attending neonatologist. As a result, only 148 of the 1445 infants (approximately 10%) included in the study underwent MRI, leading to the exclusion of 1297 infants (90%) from the analysis. This introduces potential selection bias, as those who received MRIs may not fully represent the broader population of infants receiving PN, thus limiting the generalizability of our findings. The American Academy of Pediatrics recommends routine brain MRI only for high-risk premature infants, which may further explain our smaller sample size [26, 29]. To better understand the neurodevelopmental outcomes of these infants, longitudinal follow-up across childhood stages is crucial. Tracking neurodevelopmental progress over time will help to identify the types and severity of disabilities associated with the observed structural changes.

In conclusion, we do not have the optimal composition of LEs that mimic natural and enteral lipid sources. Furthermore, we are not optimistic about the liberal use of SMOFlipid as a solitary lipid source for improving brain morphology and maturation of premature babies. A well-designed randomized controlled trial is required, and meanwhile, the use of SMOFlipid or any new generation lipid emulsion must follow strict and well-observed protocols.

## Data Availability

The datasets used and/or analysed during the current study available from the corresponding author on reasonable request.
